# Editorial: Physiology and pathophysiology of the olfactory system

**DOI:** 10.3389/fncir.2022.1025087

**Published:** 2022-10-25

**Authors:** Hideki Kashiwadani, Thomas Heinbockel, Fumiaki Imamura, Masahiro Yamaguchi, Sachiko Koyama, Kenji Kondo

**Affiliations:** ^1^Department of Physiology, Graduate School of Medical and Dental Sciences, Kagoshima University, Kagoshima, Japan; ^2^Department of Anatomy, Howard University College of Medicine, Washington, DC, United States; ^3^Department of Pharmacology, Penn State College of Medicine, Hershey, PA, United States; ^4^Department of Physiology, Graduate School of Medicine, Kochi University, Kochi, Japan; ^5^Department of Chemistry, Indiana University, Bloomington, IN, United States; ^6^Department of Otolaryngology-Head and Neck Surgery, Graduate School of Medicine, The University of Tokyo, Tokyo, Japan

**Keywords:** olfaction, olfactory cortex, olfactory bulb, olfactory epithelium, odor-guided behavior

One of the intriguing features of the mammalian olfactory system is the integration of newly generated sensory neurons and interneurons in the olfactory bulb throughout our life. The continuous reconstitution of the olfactory neural circuit that arises from the integration could modify the olfactory information processing, and thus impair the olfactory image of objects. Therefore, to maintain the olfactory perception, the olfactory system has developed homeostatic plasticity which works under physiological and even pathophysiological conditions.

This Research Topic intends to represent the most recent and exciting results focused on understanding the mammalian olfactory system covering the olfactory epithelium to higher olfactory centers, from molecular mechanisms to olfactory recognition.

We are truly thankful to all contributors to this Research Topic. Twenty-six authors contributed original research and review articles. Furthermore, we are deeply appreciative of all reviewers and editors who helped us to make an interesting, high-quality, and exciting Research Topic.

## Homeostatic mechanisms of the olfactory mucosa

In this section, two papers have described research data about the homeostasis of the olfactory neuroepithelium. Mori et al. examined whether the rat olfactory neuroepithelium fully recovers after the mucosal resection. Partial regeneration of the neuroepithelium was observed within 1 month after resection, whereas subsequent degeneration into squamous and respiratory epithelia occurred within 3 months. The authors conclude that, because of the poor neuroepithelial regeneration after the mucosal resection, surgeons should be cautious not to injure the olfactory mucosa during nasal surgery. Kikuta et al. examined the effects of insulin on the prevention of olfactotoxic drug-induced neuroepithelial injury in mice. Unilateral insulin administration prevented the methimazole-induced reduction in the number of olfactory sensory neurons on the ipsilateral side. Intranasal administration of eosinophilic cationic protein damaged the olfactory neuroepithelium by inducing apoptosis, but this damage was largely prevented by insulin administration. Therefore, insulin administration might lead to the development of new therapeutic agents for olfactory neuroepithelial injury. The information provided by these papers is valuable for our understanding of homeostasis of the olfactory neuroepithelium as well as for the development of a therapeutic strategy for olfactory dysfunction.

## Olfactory cortical circuit and information processing

Two papers on neural circuits and information processing in the olfactory system have contributed to this Research Topic. Mori and Sakano summarize the recent progress in olfactory information processing during the respiratory cycle. The authors highlight studies suggesting that the tufted cell pathway associated with learned decisions is activated during the inhalation phase, while the mitral cell pathway is stimulated during the exhalation phase for instinct decisions. Furthermore, differential processing of orthonasal and retronasal signals in separate areas of the olfactory bulb and olfactory cortex is proposed in this review. Maegawa et al. report the expression of prodynorphin (Pdyn) and preproenkephalin (Penk), precursors of endogenous opioids, in the mouse olfactory tubercle. Using the multiple-fluorescence *in situ* hybridization technique, the authors show that Pdyn and Penk are primarily expressed by cells expressing dopamine receptors D1 (Drd1) and D2 (Drd2), respectively. Furthermore, it is shown that there are Pdyn-Penk-Drd1 co-expressing cells, which are found more abundantly in the anteromedial olfactory tubercle than in the anterolateral olfactory tubercle. These two articles provide important insights into olfactory circuitry and olfactory information processing.

## Human olfaction

The olfactory system affords the ability to form an olfactory image of an object and also the ability to attend to odors. However, the neuronal circuit mechanisms underlying the attentional function in human olfaction were not yet revealed. Zhang et al. reported in this Research Topic that an unpleasant odor induced larger cue-related EEG responses in the alerting and executive control networks which correlated with a reduced behavioral response time of cue-related trials. Interestingly, these responses were irrespective of the odor concentration. These findings support ecological evidence for unpleasant odors acting as warning signals to help humans to stay alert to potential threats in the environment. The findings also provide cognitive performance evidence to support the relevant arguments of evolutionary psychology as well as situations in real-world daily life.

## Olfactory diseases

COVID-19 has had a significant impact on the chemical senses of many patients. So many COVID-19 patients experienced chemosensory dysfunction that the symptoms of chemosensory dysfunction became very well known to the public compared to the pre-pandemic time, causing an illusion that olfactory dysfunction is typical for COVID-19 but not for other diseases. In the review article by Caretta and Mucignat-Caretta, the authors discuss that chemosensory dysfunction has been observed in various diseases such as neurodegenerative diseases, neuropsychiatric diseases, dysmetabolic diseases, cancer, autoimmune disorders, cardiovascular diseases, kidney, liver, and lung diseases, and poisoning, other than infectious diseases such as COVID-19. In the case of infectious diseases, viruses other than SARS-CoV-2, bacteria, fungi, and protozoa can cause chemosensory dysfunction, and it is not unique to COVID-19. In the pre-COVID-19 era, these symptoms tended to be ignored or under-appreciated because they were considered not life-threatening. The authors discuss that “notably, dysfunction of olfaction correlates with increased mortality and with patient frailty in the general population, possibly mediated by inflammation” and suggest that “putting chemosensory dysfunction on the clinical spot may help in understanding the patient's symptoms and the underlying pathophysiology of complex diseases, involving multiple systems' integrated response”. Changes in the under-appreciated sense of olfaction could be a key sign of complex diseases that better receives more clinical attention.

## Author contributions

HK, TH, FI, MY, SK, and KK wrote the draft of the editorial. All authors revised the manuscript and approved the final version of it.

## Conflict of interest

The authors declare that the research was conducted in the absence of any commercial or financial relationships that could be construed as a potential conflict of interest.

## Publisher's note

All claims expressed in this article are solely those of the authors and do not necessarily represent those of their affiliated organizations, or those of the publisher, the editors and the reviewers. Any product that may be evaluated in this article, or claim that may be made by its manufacturer, is not guaranteed or endorsed by the publisher.

